# Testing the impact of a social skill training versus waiting list control group for the reduction of disruptive behaviors and stress among preschool children in child care: the study protocol for a cluster randomized trial

**DOI:** 10.1186/s40359-017-0197-9

**Published:** 2017-08-07

**Authors:** Sylvana M. Côté, Marie-Pier Larose, Marie Claude Geoffroy, Julie Laurin, Frank Vitaro, Richard E. Tremblay, Isabelle Ouellet-Morin

**Affiliations:** 1 University de Montréal, 3050 Édouard-Montpetit, Montreal, H3T 1J7 Canada; 2 University de Bordeaux, INSERM U1219, Bordeaux, France; 3 University McGill, Montreal, Canada; 4 University College Dublin, Dublin, Ireland

**Keywords:** Child care services, Intervention, Social skill training, Cortisol, Social development, Poverty

## Abstract

**Background:**

Most preschoolers growing up in western industrialized countries receive child care services (CCS) during the day, while their parents are at work. Meta-analytic data suggest that CCS represent a stressful experience for preschoolers. This may be because preschoolers have not yet developed the social skills necessary to cope with the new and rapidly fluctuating social contexts of CCS. We tested the effectiveness of a child care-based social skill training program aiming to improve children’s social behaviors and reduce the stress they experience.

**Method and design:**

We used a cluster randomized control trial (cRCT) to compare children’s social behaviors and stress levels in pre- and post-intervention according to whether they received a social skill training intervention or not. Nineteen (*n* = 19) public CCS (*n* = 362, 3-years-old preschoolers) of underprivileged neighborhoods (Montreal, Canada) were randomized to one of two conditions: 1) social skills training (*n* = 10 CCS); or 2) waiting list control group (*n* = 9 CCS). Educators in the intervention group conducted bi-weekly social skills training sessions over a period of 8 months. The intervention covered four topics: making social contacts, problem solving, emotional self-regulation, as well as emotional expression and recognition. Main outcome measures included preschoolers’ disruptive (e.g. aggression, opposition, conflicts) and prosocial behaviors (e.g. sharing toys, helping another child), and stress levels assessed by salivary cortisol sampling at pre and post intervention assessments. Educators’ practices will be tested as potential mediators of the expected changes in behaviors and neuroendocrine stress.

**Discussion:**

To our knowledge, this is the first cRCT to test the effectiveness of a child care based social skill training program on the reduction of disruptive behaviors and levels of stress. Significant challenges include the degree of adherence to the intervention protocol as well educators and preschoolers’ turnover.

**Trial registration:**

Current clinical trial number is ISRCTN84339956 (Ongoing study, Retrospectively registered on March 2017) No amendment to initial protocol.

**Electronic supplementary material:**

The online version of this article (doi:10.1186/s40359-017-0197-9) contains supplementary material, which is available to authorized users.

## Background

In most western industrialized countries, the use of child care services (CCS) during the preschool years increased constantly since the middle of the 1980’s [1]. It is estimated that more than three children in four receive full time CCS before they enter the elementary school system [2]. We use the term child care services (CCS) to refer to regular group-based care of children prior to school entry (i.e. under age 5 years in North America) by someone else than the parents.

Several studies show benefits of high quality CCS on social and cognitive development and school readiness, especially for children from low socioeconomic status families [3–5]. However, other studies indicated that the child care environment may also represent a source of stress, especially for 3- and 4-year-old preschoolers [6–8]. Indeed, a number of studies report an increase of cortisol from morning to afternoon among children receiving regular CCS instead of the expected decrease [9, 10], a pattern of secretion not observed at home [9].

There are two reasons that might explain why preschoolers aged between 3 and 4 years are more likely to show a disrupted pattern of circadian cortisol secretion while they are in CCS. First, this period coincides with peak levels of physically aggressive behaviors in response to conflicts, thereby increasing the probability of being the victim of or manifesting physically aggressive acts while doing group activities [11]. Being involved in conflicts involving physically aggressive behaviors is considered a major stressor in both animal and human stress studies [12, 13]. Second, when children reach the ages of 3–4 years, there is a normative increase in the quantity of interactions with their peers and a corresponding decrease in parallel play [6, 9, 14]. In addition, child care activities become increasingly oriented toward interactive games. Thus, there is an unprecedented demand for social interactions [7]. However, preschoolers have not reached a sufficient level of emotional control [7], behavioral skills [14], and language development [15] to exhibit the social skills necessary for un-stressful social interactions. Hence, there is a gap between, on one hand, their desire and the contextual pressure for social interactions and, on the other hand, their actual social and cognitive capacities to interact with each other. These observations could explain why preschoolers in child care have higher cortisol pattern than children who stay-at-home.

The child care context requires the ability to exhibit basic social skills and children with behavior problems may be particularly likely to experience high levels of stress. Indeed, children with high levels of aggressive behaviors, low levels of social competence [7], and who suffer from peer rejection are those exhibiting the highest levels of cortisol [10]. Children from lower socio-economic status (SES) families are particularly at risk of exhibiting disruptive behaviors during the preschool years and therefore to experience stressful CCS experiences. Low SES is associated with higher risk of school difficulties and school failure during middle childhood and adolescence [16–18], but CCS can buffer this risk [4, 19]. Thus, intervention aiming at improving social skills and reducing disruptive behaviors in CCS of lower SES neighborhoods will reach a larger proportion of children at risk that could potentially benefit from a Child care based social skill training.

A recent meta-analysis concluded that psychosocial interventions based on behavioral-cognitive strategies were effective for the reduction of children’ disruptive behaviors [20] and that interventions conducted during early childhood might be more effective on the long term for psychosocial outcomes than those conducted during middle childhood or adolescence [20]. Most interventions focused on children directly and individually, although, child care services might be a promising setting for group-based prevention [21]. Notably, we are not aware of intervention who have documented the impact of child care-based prevention programs on preschoolers’ levels of stress, even though psychosocial interventions showed promising results to improve stress regulation among children of this age group [22].

### Objectives

The aim is to test the effectiveness of a social skill training interventions aiming at improving social behaviors among 3 year-old children in CCS of low-SES neighborhoods using a cluster Randomized Control Trial (cRCT). Children attending CCS where the program was implemented the first year of the study were compared to those attending CCS on a waiting list control group (program implemented the second year).

The ‘Minipally’ social skill program is a 16-week intervention supporting the development of social and self-regulation skills among children aged 2 to 5 years. The program was delivered by child care educators who receive a 2-day training and regular supervision during the program.

The trial included two primary outcomes: children’s social behaviors (i.e. aggression, opposition, impulsivity, prosociality) and children’ stress levels (cortisol diurnal circadian rhythm). Both types of outcomes were measured before and at the end of the 8-months intervention. We hypothesized that the Minipally program would improve preschoolers’ social skills and, preschoolers’ stress regulation. A secondary objective was to assess the impact of the intervention on educator’ practices.

## Methods/designs

### Design

The Minipally Study is a ongoing, prospective, superiority, cluster-randomized controlled trial (cRCT), with two parallel arms comparing children attending CCS where the social skill training intervention was implemented in year 1 with those receiving the intervention in years 2 (waiting list control group). The trial used an 8 months’ pre-post single blind (i.e. blinded evaluators) methodology with pre- (T1) and post-intervention (T2) assessments.

### Study setting

**Fig. 1:**
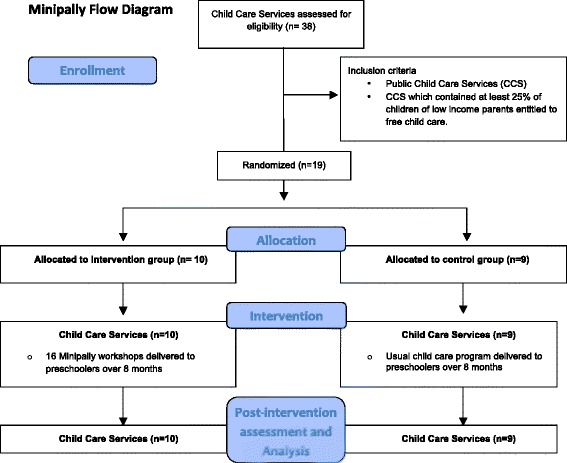
Minipally flow diagram

This study took place in public CCS in the province of Quebec in Canada. Directors of *n* = 38 public CCS of the greater Montreal region located in low SES neighborhoods were invited to participate in a study on the impact of a social skill training program on children’s social behaviors and levels of stress. CCS eligibility criterion for participation was limited to those with a minimum of 25% of children from low-income families. Low income families were those entitled to a special provincial subsidy program providing free child care access, representing a annual familial income below 20,000 can$. Nineteen CCS met eligibility criteria and were include in the trial. The flow chart of the randomization process is presented in Fig. 1.

### Randomization and masking

CCS were randomly assigned in a 1:1 ratio to either the Minipally intervention in year 1 or waiting list control using a computer-generated randomization sequence. In accordance with PROBE methodology, the team of investigators, including Research Assistants (RAs), was blind to the assignment of the CCS in the two conditions during the study. However, CCS directors, educators as well as parents were aware of group membership after randomization, as per the open-label design.

### Interventions

Minipally is a social skills training program supporting the development of adaptive social behaviors among children aged 2 to 5 years attending group-based CCS. It includes generic components of social skill training programs: introduction to social contact (make and accept contact from others, make requests); problem solving (identifying the problem, generating solutions); self-regulation (breathing to calm down, accepting frustration, learning to share, tolerating frustration); and emotional regulation (identifying and expressing emotions, listening to the other). The program is delivered via 16 playful sessions animated by the educators over a period of 8 months. Minipally is a puppet who becomes a loyal and enthusiastic friend of children, visits them every two weeks and takes advantage of his visits to model prosocial behaviors and promote social inclusion by discussing/playing with his friends (other puppets) and with the children. Minipally was adapted from a social skill training programs for school-aged children (i.e. Fluppy program) shown to have long-term effectiveness on academic achievement, employment, income, delinquency and substance abuse [17, 23]. Over the past 20 years, experienced educational psychologists and psychoeducators have updated the Fluppy program to address 1) the evolution of best practices in social skill training and 2) adaptation to younger age groups, i.e. preschool aged children. The Minipally program is the result of these adaptations. Training in the use of Minipally was offered to child care educators for more than 5 years prior to the study. However, the efficacy of program, unlike its version for older children (Fluppy), had never been formally evaluated.

In the present study, the Minipally implementation followed the same procedure as that used in a non-research environment. That is, a team of experienced psychologists and psychoeducators who adapted the program to preschool children provided Minipally training to the educators. Each educator had two training days and 12 h of supervision within the three first months of the intervention.

### Description of the control group: Child Care Services (CCS) assigned to waiting list

Educators working in the CCS randomly assigned to the waiting-list control group did not receive any training the first year, so that children in their group were exposed to usual educational practices. At the end of year 1, educators in the control CCS received the Minipally training program.

### Adherence and withdrawal

#### Intervention’ adherence

The Minipally program was part of the educational practices to which all children in the participating CCS were exposed. Parents were informed of the Minipally program via a letter sent by the head of the CCS. Parents could refuse the participation of their child in the pre and post intervention assessments (saliva samples to measure cortisol and behavioral assessments by the educator), but all children in the group were exposed to Minipally activities.

Educators in the intervention group completed a logbook in which they indicated which activity was conducted in their classroom and the date of the activity. In situation of staff turnover in the intervention group, an attempt was made to provide training to the new educator. However, in some cases this was not possible due to time constraints (lack of time by the educator or too late in the school year). The impact of the intensity of exposure to the intervention will be examined.

#### Evaluation’ adherence

For children who were absent on the day of the CCS visit in pre or post-intervention, preschoolers’ saliva could not be sampled. However, educators and parents rated behavioral questionnaires about the child on a day when he was present and this information was used in the analyses. In order to optimize adherence to the evaluation by the educators and parents, CCS were called to remind their educators and the parents of upcoming visits of Research Assistants (RAs).

### Procedure and measures

Outcomes were assessed during two visits to the CCS – one at pre (T1 in October) and one at post intervention (T2 in June). A team of two trained research assistants, blinded to intervention assignment status of the CCS, was present early in the morning (approximately 7 h30 am) to collect the first cortisol sample of the day (30 min after arrival at CCS), and to give the behavioral and general health questionnaires to parents at CCS arrival. Research assistants stayed all day at CCS to perform the different assessments until the last cortisol sample was taken (i.e. 30 min after the nap, at about 14 h–16 h). Research assistants also asked the educators to complete behavioral questionnaires about every child in their group. All the outcome measures and their times of assessment are presented below and in Table 1.

**Table 1:** Summary of instruments used in the Minipally study

Outcome measures			
Concepts	Type and Source of measure	Instrument	Time of assessment
Primary outcomes			
Child Behaviors	Questionnaire, rated by childcare’ educators Questionnaire, rated by parents	Child Behavior Questionnaire (BEH, 24)	Pre and post-intervention
Opposition			
Hyperactivity			
Prosociality			
Inattention			
Agression			
Child’ agressive and prosocial behaviors	Observed and rated by research assistant	Observational tool designed for the study	Pre and post-intervention; at Child Care Services
Stress	Salivary cortisol, collected by research assitant	Biological samples	Pre and post-intervention; at Child Care Services
			Child Care Arrival (7 h30-10 h)
			Before Lunch (10 h30-12 h)
			30 min after napping in the afternoon (14 h–16 h)
Secondary outcomes			
Educational interactions with the children	Observed and rated by research assistant	Caregiver Interaction Scale (29)	Pre and post-intervention; at Child Care Services
Severity			
Detachment			
Permissiveness			
Harshness			
Educator’s behaviors with each child	Questionnaire, rated by childcare’ educators	Pacotis (30)	Pre and post-intervention
Potential confounding variables			
Sociodemographic information	Questionnaire, rated by child’ parents	Questionnaire designed for the study	Pre and post-intervention
Child’ General Health	Questionnaire, rated by child’ parents	Questionnaire designed for the study	Pre and post-intervention
Child’ Stressful Life Events	Questionnaire, rated by child’ parents	Stressful Life Event Questionnaire	Pre and post-intervention

### Primary outcome measures

#### Children’s Behaviors assessed by child care educators and parents

The educators completed the BEH questionnaire [24] for each child in his or her group. Each parent also completed the BEH for his child before and after the intervention. The 25-item questionnaire covers the five behavioral dimensions: Opposition (5 items, e.g. has been defiant or has refused to comply to adults request); impulsivity/hyperactivity (4 items, e.g. has had difficulty waiting for his/her turn in games); physical aggression (6 items; 3 reactive, e.g. has reacted in an aggressive manner when teased, 3 non-reactive, e.g. has gotten into fights); pro-sociality (7 items, e.g. has helped other children); and inattention (3 items, e.g. has been easily distracted, has had trouble carrying out any activity). The questionnaire has adequate psychometric properties (Cronbach alpha = .86; test-retest reliability varies from .76 and.86) [24]. Items of the BEH used in the Quebec Longitudinal Study on Child Development (QLSCD) incorporated items from Preschool Behavior Questionnaire [24, 25], Child Behavior Checklist [26], and Strengths and Difficulties Questionnaire [27].

#### Frequency of conflicts and prosocial behaviors assessed by research assistants

Two research assistants independently coded an 8-item observational grid to assess the frequency of conflicts and prosocial behaviors exhibited in the classroom during a 15-min free play activity period. Three forms of aggressive behaviors were coded: aggression toward another person; aggression toward an object; and verbal aggression. Aggressive behaviors towards another person refers to any physically aggressive behaviors that might harm another person including hitting, pushing, hair pulling, toy grabbing, throwing objects directly at someone. Aggressive behaviors towards an object included destruction of objects; hitting or kicking a toy or object; throwing an object on the ground or at the wall. Verbal aggression concerned any threats or verbal intimidation exhibited by children, including screaming after or insulting another child. Finally, five prosocial behaviors included sharing a toy, inviting a friend to play, helping out a friend in need, requesting help from an adult, and requesting an object. The number of times that each of these eight behaviors was exhibited in the classroom was collected in a tally system for the whole classroom’s free play period, thus providing a group-level assessment of social behaviors. A similar observational procedure to ours is found to provide reliable assessments of social behaviors in group contexts among children in this age group [28].

#### Levels of stress assessed by salivary cortisol collected by research assistant

Salivary cortisol samples were collected by research assistants three times during the CCS visit, at both pre and post intervention: 1) 30 min after the child’s arrival (between 7:30 and 10 am); 2) before lunch (between 10:30 and 12:00); 3) 30 min after waking from the afternoon nap (between 14:00 and 16:00). One ml of saliva was collected with a narrow cotton sponge (diameter: 8 mm) covered with a thin, perforated plastic film (sterile and packaged individually). The sponge’s elongated form allowed the research assistant to hold the other end of the cotton sponge with a sterile glove. The sponge was kept under the child’s tongue for a minute. Salivary samples were then stored at −20 °C until cortisol concentration determination. Laboratory analyses were performed using a high sensitivity Enzyme Immunoassay (Salimetrics, LLC from State college, PA, USA). The lowest limit of detection is 0.007 μg/dL, and all samples were assayed in duplicates.

### Secondary outcomes

#### Educators’ interactions with children assessed by research assistant

One of the research assistant completed the Caregiver Interaction Scale [29] to assess the extent to which educators exhibited behaviors classified along four dimensions: sensitivity, harshness, detachment, and permissiveness, using a scale ranging from 1 (did not exhibit) to 4 (exhibited often the selected behavior).

#### Educators’ interactions with children assessed by child care educators

Educators rated the frequency of positive and negative interactions with each child in their group at both pre and post-intervention using a subset of the PACOTIS questionnaire [30]. The 9-item questionnaire relies on a 5-point Likert scale (1: never to 5: all the time) to rate the frequency of educator’ positive interactions (i.e. comfort, play, laugh, monitor misconduct) and negative interactions (i.e. threaten punishment, coercion) with each preschooler.

### Potential confounders

#### Sociodemographic information assessed by parents

Information about parents’ socio-demographics (education and income) was collected at pre-intervention for all children. With the socio-demographic questionnaire, we collected information about children’ CCS attendance such as the number of hours children attend CCS per week and the number of months the child attends a CCS.

#### General health questionnaire assessed by parents

Parents filled out a short questionnaire in pre and post-intervention upon CCS arrival to obtain information about a wide range of factors known to potentially affect cortisol secretion, including the child’s general health (infection, allergies, temperature, a tooth or ear ache, fracture or a sprain); sleep quality during the previous night (bedtime, wake-time, night-waking length, quality of sleep); his/her mood at the arrival to the CCS (fatigued, happy, sad, worried, excited, angry); chronic or serious health concern (juvenile diabetes, asthma, cardiac problems, had undergone surgery or been hospitalized in the last year); use of prescription or non-prescription drugs in the last 24 h; and the food consumed in the morning of the assessment (time of breakfast, quantity of dairy product consumption, time of snacks taken after breakfast) [31].

#### Stressful life events assessed by parents

A subset of the Stressful Life Events Questionnaire [32] was selected for the present study. Parents were asked in pre and post-intervention whether 13 stressful life events (e.g., birth, death, illness/accident, employment, financial and family status changes, family move, and trouble with the law) have occurred in the past 12 months. For each stressful event, parents were asked about the occurrence of the event (0: “no” to 1: “yes”) for each family member (i.e. biological mother and father, sibling, grand-parents). Parents were also asked to evaluate the perceived valence and intensity of this stressful event on their child (5-point likert scale: −2: extremely negative to 2: extremely positive). A cumulative score of stressful events exposure was created to assess child socio-environmental adversity.

### Statistical analysis

#### Determination of sample size

The trial was designed to test whether the Minipally intervention was superior to the usual educational in terms of stress regulation and children’ behaviors. We used Heo’s statistical procedure for cluster randomized trial in our sample size estimation [33]. That is, we based our calculation on the expected mean number of groups within each child care centers –i.e. 2 groups per child care center. Recall that the CCS are our randomization units. Power calculation indicated that 19 child care services would allow to detect a medium size effect of the intervention on the selected outcomes, with 90% power at a 2-sided significance level of α = 5%. Our model can be stated as Y_ijk_ = β_0_ + δX_i_ + u_i_ + u_j(i)_ + e_ijk_; where Y_ijk_ is the post-intervention response of the i^th^ study participant in the j^th^ educator group nested the k^th^ child care services, β_0_ correspond to baseline value of our primary outcome, δX_i_ assess the main effect of the intervention (where X = 0 for waiting list group and X = 1 for the experimental group), and the last three terms are random effects at every level of the trial analysis [33].

Data will be analyzed per the intention-to-treat principle where all participants are considered within their assignment group regardless of the number of Minipally sessions they received. Every child whose parents consented to the evaluation will be included for analysis if they completed at least one assessment (i.e. pre or post-intervention).

### Data analysis

#### Testing the equivalence of the intervention and control groups at baseline

We will compare the intervention and control groups on a wide range of family, health and child variables assessed at pre-intervention to test whether the randomization procedure was successful in yielding equivalent groups. This is a first step in examining the extent to which biological and behavioral impacts observed in post-intervention are associated with the Minipally intervention, and not to confounding differences at baseline between treatment groups. Specifically, group differences will be examined for socio-demographic information (age, income and education of the parents, household composition); child variables such as the rates of disruptive behaviors; and CCS related variables such as number of hours attending child care services per week, number of months attending a child care service. If differences are detected, these variables will be statistically controlled for in subsequent analyses.

### Testing the impact of the intervention

#### Child behaviors

We expect that children in the Minipally intervention condition will have better social behaviors -higher levels of prosocial behaviors’ and lower levels of opposition, hyperactivity, inattention and physical aggression disruptive behaviors- as compared to children in the waiting list control group in post-intervention. We will use multilevel models where the child’ behaviors in pre-intervention will be considered as a covariate. We will thus 1) account for initial variation in children’s behaviors, and 2) account for the nested structure of the data: children are nested in childcare’s group (i.e. *n* = 8 children per educators) and the childcare’s groups are nested in child care services (i.e. *n* = 1 to 4 groups of preschoolers per child care services).

### Stress regulation

We hypothesized that children receiving the Minipally program will have better levels of stress regulation (i.e. better diurnal salivary cortisol patterns) at the end of the 8 months intervention than children in the waiting list control group. To model the impact of intervention on preschooler’ stress level, we will use a growth curve model accounting for time variation in cortisol, as cortisol samples were taken three times a day (at the arrival to the CSS, midmorning and 30 min after the afternoon nap). We expect to find a significant difference between the mean slope of cortisol secretion between the intervention and the control group in post-intervention, but not in the intercept levels. Growth curve models also account for the non-independence of repeated measures by modeling multiple data points as nested within individuals, which further allows for missing data. This technic accounts for shared variance within subjects while modeling between-subject differences. The normality assumptions for all our potential covariates and models will be assessed using standard statistical methods and comparison of residual versus predicted plots and residual versus normal scores.

Note that prior to any impact analyses, we will examine the cortisol data for extreme values. Cortisol outliers will be winsorized at >3 standard deviations ud/dL to ensure that extreme values do not exert a disproportionate influence on analysis. Cortisol values have skewed distribution and therefore will be transformed using Log 10. A constant of 1 will be added to original concentration [Log 10 (concentration + 1)] to assure positive transformed cortisol data. We will then use univariate and multivariate models to assess several factors known to potentially affect cortisol secretion (e.g. general sleep, general health, medication) and control for them in later analyses.

### Secondary analysis

Analysis of mediator and moderator of the putative impact of the intervention will be conducted. Potential mediators include change in educators’ educational practices or behaviors in the classroom as a consequence of the program. Potential moderators include family socioeconomic status, intensity of the intervention or characteristics of the educators such as his/her level of training or age. These analyses will provide information on potential routes via which the intervention has an impact (mediation) or potential subgroups, which benefited more or less from the intervention (moderator).

### Ethical principles and safety

Consents to participate in the study were obtained from parents, educators and head of the CCS. The Sainte-Justine Hospital Ethical Research Committee approved all procedures in May 2013 ref.: 2014–565, 3738. A renewal of the ethic approval is delivered every year since then. The data were stored on a confidential server hold in the Sainte-Justine Hospital. Additional file 1 includes consent forms and ethical approval certificates.

## Discussion

The Minipally study is the first cluster randomized-controlled trial to test the effectiveness of a child care-based social skills training program to improve social behaviors and reduce levels of stress among preschool children. A specificity of the program was its assessment amongst CCS of low SES neighborhoods.

This trial is innovative as few studies have experimentally tested the hypothesis that a social skill training program in CCS could improve children’ behaviors and reduce children’s levels of stress. One similar intervention to ours is The Dinosaur Program from the Head Start Project in United States [16]. The Dinosaur Program was a prevention curriculum that aims to increase social, emotional and academic competences among young children in kindergarten [16]. With the Minipally program, we propose to intervene even earlier (i.e. with preschoolers in child care services) on psychosocial functioning and on stress regulation, as the ability to manage emotional arousal and to make meaningful friendship is an important aspect of children's optimal development.

There are two main reasons why the timing of the Minipally intervention is promising. First, the preschool years are a developmental period where preschoolers establish long-term physiological processes and parameters [34]. This issue reinforces the need to intervene on stress regulation in child care as the preschool years might be the most effective period to set reactive parameters to stressful encounters. Second, the preschool years represent a particularly socially demanding period for children in CCS as they are not well equipped to deal with the plurality of social challenges. Hence, by teaching social skills and self-regulation strategies to children in CCS, ﻿Mninipally ﻿aims at facilitating children’s adaptation to the group context.

One of the main strength of this cluster randomized trial is its relatively large sample size (n CCS = 19, n children = 362) and the quality of the outcomes measurements (i.e. 3 cortisol samples per day in pre and post-intervention), as well as its multi-informant design to assess child social skills (i.e. observational data from research assistant, questionnaire from parents and child care’ educator). To our knowledge, no previous child care intervention study relied on an cluster experimental design (i.e. randomized child care services) to study social skills improvement and stress regulation among children. This design allows for both methodolical rigor and the respect of ethical RCT guidel﻿ines.

Several studies suggest that child care services of sufficient quality ﻿may be effective in promoting cognitive and social development, essential components of school readiness [2–4, 16]. A pedagogical program promoting social skills, if shown to improve social behaviors, would provide information on useful strategies to improve CCS. This knowledge is needed to identify how to best invest in early childhood, considering that early childhood investments present better returns compared to investments later in life [35].

## Additional file

Additional file 1: Ethical and consent forms. (DOC 121 kb)

## Abbreviations

CCS: Child care Services
GRIP: Research unit on children’s psychosocial maladjustment
SES: Socioeconomic background
